# Inhibition of virulence potential of *Vibrio cholerae* by natural compounds

**Published:** 2011-02

**Authors:** Shinji Yamasaki, Masahiro Asakura, Sucharit Basu Neogi, Atsushi Hinenoya, Emiko Iwaoka, Shunji Aoki

**Affiliations:** Graduate School of Life & Environmental Sciences, Osaka Prefecture University, Osaka; * *Faculty of Pharmacy, Hyogo University of Health Sciences, Kobe, Japan*

**Keywords:** Capsaicin, cholera toxin, real-time PCR, spice, *Vibrio cholerae*

## Abstract

The rise in multi-drug resistant *Vibrio cholerae* strains is a big problem in treatment of patients suffering from severe cholera. Only a few studies have evaluated the potential of natural compounds against *V. cholerae*. Extracts from plants like ‘neem’, ‘guazuma’, ‘daio’, apple, hop, green tea and elephant garlic have been shown to inhibit bacterial growth or the secreted cholera toxin (CT). However, inhibiting bacterial growth like common antimicrobial agents may also impose selective pressure facilitating development of resistant strains. A natural compound that can inhibit virulence in *V. cholerae* is an alternative choice for remedy. Recently, some common spices were examined to check their inhibitory capacity against virulence expression of *V. cholerae*. Among them methanol extracts of red chili, sweet fennel and white pepper could substantially inhibit CT production. Fractionation of red chili methanol extracts indicated a hydrophobic nature of the inhibitory compound(s), and the n-hexane and 90 per cent methanol fractions could inhibit >90 per cent of CT production. Purification and further fractionation revealed that capsaicin is one of the major components among these red chili fractions. Indeed, capsaicin inhibited the production of CT in various *V. cholerae* strains regardless of serogroups and biotypes. The quantitative reverse transcription real-time PCR assay revealed that capsaicin dramatically reduced the expression of major virulence-related genes such as *ctxA, tcpA* and *toxT* but enhanced the expression of *hns* gene that transcribes a global prokaryotic gene regulator (H-NS). This indicates that the repression of CT production by capsaicin or red chili might be due to the repression of virulence genes transcription by H-NS. Regular intake of spices like red chili might be a good approach to fight against devastating cholera.

## Introduction

Cholera is an acute diarrhoeal disease which is characterized by discharge of voluminous rice water stool caused by toxigenic *Vibrio cholerae* strains. *V. cholerae* O1 and O139 serogroups producing cholera toxin (CT) are mainly responsible for cholera outbreaks that can cause havoc in highly populated regions in Asia, Africa, and Latin America^1^. *V. cholerae* O1 is further divided into El Tor and classical biotypes. The ongoing pandemic of cholera that started in 1961 is caused by O1 El Tor biotype, which replaced O1 classical strains that caused previous six pandemics. The O139 serogroup evolved as a new epidemic strain in 1992^2^. Currently, the El Tor variant strains possessing classical type ctx genes are mainly responsible for cholera outbreaks in many developing countries^3^. Strains of *V. cholerae*, which do not belong to O1 or O139 serogroups are commonly called as non-O1/non-O139, some of them are toxigenic and can cause sporadic cases of diarrhea^4^. In serious cases, the infection can produce an acute state of dehydration within several hours and if untreated, can be fatal. Among various virulence factors reported in *V. cholerae*, CT (encoded on a filamentous phage called CTX-Φ) consisting of one A and five B subunits and the toxin co-regulated pilus (TCP) are the most important factors associated with severity of the illness. TCP can act as a receptor for CTX-Φ and also aid in bacterial colonization in our intestine^5^.

Oral rehydration solution (ORS) is a commonly used therapy for cholera that aids recovery from dehydration. However, due to the severity of the disease, antimicrobial agents are most often administered in addition to intake of ORS. Unfortunately, through mutation and the selective pressure exerted by the antimicrobial agents, *V. cholerae* strains have been increasingly becoming resistant to commonly used antimicrobial agents. In the past two decades, most of the *V. cholerae* strains in cholera endemic countries have become resistant to many antimicrobial agents including tetracycline, ampicillin, nalidixic acid, streptomycin, sulphonamides, trimethoprim, gentamicin, ciprofloxacin **etc**.^6^–^8^. The emergence and spread of multi-drug resistant (MDR) pathogenic bacteria have created the need for the development of novel therapeutic agents. Conventional antimicrobial agents are generally bacteriocidal or bacteriostatic which may foster the development of MDR strains. Alternatively, inhibiting bacterial virulence factor by natural compounds is a new approach to overcome increased antimicrobial resistance in pathogenic bacteria. Commonly used natural products like spices, herbs, fruits, *etc*. have many kinds of beneficial effects to our health. In India, *Ayurveda*, a comprehensive medical system that utilizes natural products, has been used to cure many diseases including diarrhoea since ancient time. Spices and herbs have also been traditionally used to treat diarrhoeal diseases in other parts of the world. Natural products have less side effects and thus advantageous for therapeutic purpose. Recently, we have observed that extracts from some common spices can repress CT production in *V. cholerae*. Natural compounds, which can directly inhibit virulence gene expression in *V. cholerae*, may be used along with intake of ORS for treating devastating cholera.

## Plant products as remedy for diarrhoea

The beneficial health effects of many plants, used for centuries as seasoning agents in food and beverages, have been claimed for not only preventing food deterioration but also acting as antimicrobials against pathogenic microorganisms. A few studies have been carried out in a systematic manner, although phytochemical and pharmacological investigations of several plants have already led to the isolation of some of the natural antimicrobials^9^. Scientists are searching for natural products that can be used in large scale to reduce diarrhoea caused by vibrios. Some natural compounds have been examined to act against bacterial growth whereas little is known about specific influence on their virulence regulation.

The use of natural products as medicine has dramatically increased in the last two decades. Wasabi (*Wasabi japonica*), a traditional spice used with raw fish (e.g., sushi) in Japan, has been shown to have antimicrobial effect against some bacteria including *V. parahaemolyticus*^10^. Among the natural compounds possessing antimicrobial activity, a few have been tested against *V. cholerae*. Japanese green tea (*Camellia sinensis*) has been shown to inhibit both the growth and CT expression of *V. cholerae* in experimental animal^11^. Some plant polyphenols (*e.g*., procyanidins, gallate analogues, apelphenon, *etc*.) can also suppress CT activity in rabbit ileal loop or by repressing its binding to the Vero and CHO cells^12^–^15^. However, these polyphenols can act on the purified or extracellular CT but not on virulence expression of *V. cholerae*. Extracts from ‘neem’ (*Azadirachta indica*) and ‘elephant garlic’ have also been shown to inhibit *V. cholerae* growth^16^,^17^. However, any kind of antimicrobial agent targeting bacterial viability may impose selective pressure facilitating development of antimicrobial resistance. Red bayberry has the potential to inhibit the CT production in *V. cholerae* at sub-bacteriocidal concentration^18^. We have recently identified that red chili and one of its active compounds called capsaicin can inhibit CT production in *V. cholerae* without affecting bacterial growth^19^. This kind of compounds inhibiting virulence expression may impose less selective pressure on the development of antimicrobial resistance^20^. A list of natural compounds so far identified to act against diarrhoeagenic vibrios is shown in Table I.

**Table I:** Natural compounds identified to act against diarrhoeagenic vibrios

Plant	Scientific name	Specific compound	Target	Mechanism	Reference
Wasabi	*Wasabi japonica*	Allyl isothiocyanate	*V. parahaemolyticus*	Inhibit growth	10
Green tea	*Camellia sinensis*	Catechins	*V. cholerae*	Inhibit growth and CT activity	11
Guazuma	*Guazuma ulimifolia*	Procyanidins	*V. cholerae*	CT activity	12
Daio (Kampo formulation)	*Rhei rhizoma*	Gallate analogues	*V. cholerae*	CT activity	13
Apple	*Malus spp*.	Apelphenon	*V. cholerae*	CT activity	14
Hop	*Humulus lupulus*	Procyanidins	*V. cholerae*	CT activity	15
Neem	*Azadirachta indica*	Unknown	*V. cholerae*	Inhibit growth	16
Elephant garlic	*Allium ampleloprasum*	Oil (diallyl sulphides)	*V. cholerae*	Inhibit growth	17
Red bayberry	*Myrica rubra*	Unknown	*V. cholerae*	Inhibit CT production	18
Red chili	*Capsicum annum*	Capsaicin	*V. cholerae*	Inhibit CT production	19

## Inhibition of CT production in *V. cholerae* by spice extracts

Some spices (*e.g*., red chili, sweet fennel, pepper, cassia bark, *etc*.) are known to act as anti-inflammatory, anticancer, antimicrobial or antifungal agents^21^,^22^. However, there is very limited knowledge on the effect of spices on virulence regulation in pathogens. Ginger has been reported to block the heat-labile enterotoxin in *Escherichia coli*^23^. We have recently examined whether some spices contain any compound which can inhibit the virulence expression of various *V. cholerae* strains, in particular, recently emerged *V. cholerae* O1 El Tor variant^19^. Some culinary spices, which are commonly available in cholera endemic countries in South East Asia and age cost-effective were selected for this purpose.

Initially, four *V. cholerae* El Tor strains belonging to the recently emerged El Tor variant were tested against methanol extracts of red chili, sweet fennel, white pepper, red pepper, cassia bark and star anise. Spices were ground to fine powder and extracted with 99.9 per cent methanol and added to bacterial culture after appropriate dilutions. The selected strains were cultured at 37°C in AKI medium, *p*H 7.4^24^ with and without methanol extract of each spice. Cell free culture supernatant (CFS) was prepared by centrifugation followed by filtration through 0.22 μm filter. CT was purified as described previously^25^ and used as a control to quantify the concentration of CT in cultures using a previously established bead enzyme-linked immunosorbent assay (bead-ELISA)^26^. Before CFS preparation, each culture was serially diluted with phosphate buffer saline (PBS) and spread on Luria Bertani (LB) agar (Difco, KS, USA). Numbers of colonies were counted after overnight incubation at 37°C. It was observed that all the spices extracts (each 100 μg/ml) had high inhibitory activity against CT production in most of the cases (Table II). Red chili, sweet fennel and white pepper showed comparatively higher inhibitory effect (>80%) against CT production in *V. cholerae* strains than other spices^19^. The inhibitory effect on CT production in *V. cholerae* varied from strain to strain, e.g., 45-86 per cent inhibition with cassia bark and 53-80 per cent inhibition with red pepper extracts. Star anise extract had lower inhibitory effect (6-66%) on CT production when compared to other spice extracts (our unpublished data). The spice extracts (100 μg/ml) did not show any impact on bacterial growth during 1, 2, 4 and 8 h sampling time (data not shown).

**Table II:** Inhibition (%) of CT production in *V. cholerae* O1 El Tor variant strains (isolated from cholera patients in India) with six different commonly used spices methanol extract (100 μg/ml)

Strain ID	Isolation year	Red chili	Sweet fennel	White pepper	Red pepper	Cassia bark	Star anise
CO533	1994	97	95	86	68	45	50
CRC27	2000	97	92	99	80	79	66
CRC41	2000	90	96	94	53	86	6.0
CRC87	2000	94	85	87	56	78	29

*Source*: unpublished data

**Fig. 1:**
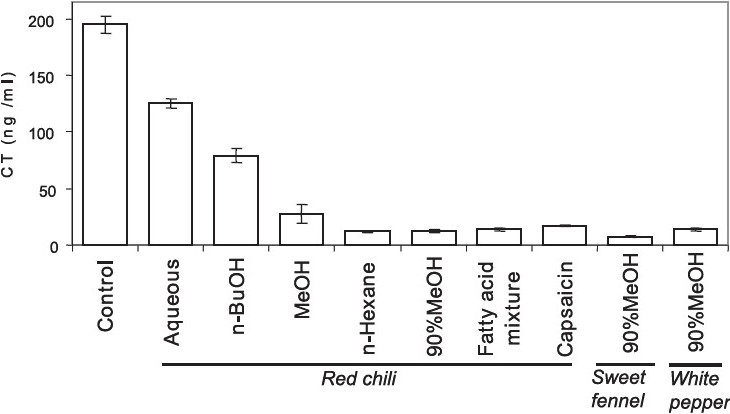
Effect of spices extracts and its purified compounds on cholera toxin production of *V. cholerae*. *V. cholerae* O1 El Tor variant strain CRC41 was cultured in AKI medium in the presence of extracts of various solvents from red chili methanol extract. Identified compounds (capsaicin and fatty acid mixture) after fractionation (by thin layer chromatography) of red chili methanol extract were also tested. Comparative inhibition of CT production by methanol extracts of sweet fennel and white pepper are also shown. The amount of CT production is represented by mean ± SD.

Among the six spices, red chili methanol extract showed potential inhibitory effect against CT production in *V. cholerae*. Red chili is an easily available natural spice and commonly used in cholera endemic regions like India and other countries in South-East Asia and Africa. The red chili extract was fractionated to purify and identify the active compounds, firstly, with aqueous/ethyl acetate mixture, then the aqueous extract was further fractionated with n-butanol while the ethyl acetate extract was further fractionated with n-hexane and 90 per cent methanol. When all these extracts were applied to the culture of a representative El Tor variant strain (CRC41), most of the inhibitory activity against CT production was detected in n-hexane and 90 per cent methanol extracts although weak inhibitory activity was also observed in other extracts (Fig. 1). The nature of such compounds responsible for CT inhibitory effect could be hydrophobic. Similarly, 90 per cent methanol extracts of sweet fennel and white pepper also showed high inhibitory effect on CT production (our unpublished data). Further fractionation of n-hexane and 90 per cent methanol extracts of red chili by silica gel and thin layer chromatography revealed that the fractions with strong inhibitory activity against CT production contained capsaicin^19^, a major component of red chili and mixture of fatty acids (our unpublished data). The inhibitory effects against CT production by both of these components were verified (Fig. 1). It has been reported that some fatty acids or their derivatives from microorganisms or plants are also sources for antimicrobial agent^27^,^28^ against Gram-positive as well as Gram-negative bacteria^29^. Recently, the role of unsaturated fatty acid from bile source to inhibit CT production in *V. cholerae* classical strain has been illustrated^30^.

## Efficacy of capsaicin as inhibitory agent against CT production

**Fig. 2:**
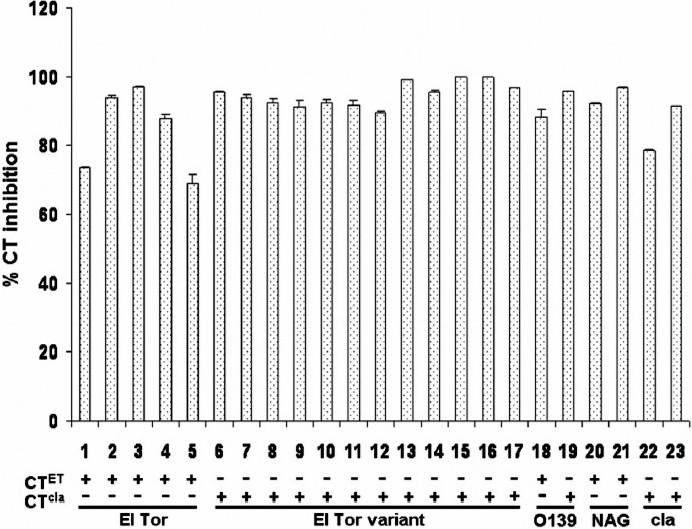
Effect of capsaicin on inhibition of cholera toxin production (%) in various *V. cholerae* strains^19^. The numbers below X-axis indicate strains belonging to O1 El Tor possessing *ctxB* of El Tor type (n=5), O1 El Tor variant possessing *ctxB* of classical type (n=12), O139 strains possessing *ctxB* of El Tor (n=1) and classical type (n=1), respectively, non-O1/non-O139 (NAG) possessing *ctxB* of El Tor type (n=2), O1 classical (cla) possessing ctxB of classical type (n=2). Each experiment was done in triplicate and the variation in CT inhibition is represented by mean × SD. ‘CT^ET^’ and ‗CT^cla^’ represent El Tor and classical type of CT, respectively

Capsaicin (N-anillyl-8-methyl-nonenamide) is one of the active ingredients in red chili which can act as an antimicrobial agent against bacterial pathogens such as *Bacillus* spp., *Helicobacter pylori*, *etc*.^31^,^32^. Besides, use of natural compounds like red chili or its derivatives like capsaicin as food supplement is advantageous because it is not harmful to common flora in human intestine^32^. Recently, we have extensively studied inhibitory activity of capsaicin on CT production in *V. cholerae* strains collected in various countries and years from 1948 to 2006^19^. Twenty three *V. cholerae* strains belonging to various serogroups and CT genotypes were selected. The ctxB genotyping of the selected strains was performed by mismatch amplification mutation (MAMA)-PCR assay^33^. It was observed that capsaicin could inhibit CT production in all kind of toxigenic *V. cholerae* strains, *i.e*., strains belonging to El Tor variant, El Tor, classical, O139 as well as non-O1/non-O139 (Fig. 2). Furthermore, we also observed that capsaicin inhibited CT production in a dose-dependent manner. Capsaicin could inhibit CT production strongly in all strains without affecting their growth, *e.g*., in case of El Tor strains (n=5) the inhibition was 70-99 per cent, in case of the recently emerged El Tor variant strains (n=12) producing classical type CT it was 90-99 per cent, in case of classical strains (n=2) it was 78-90 per cent, in case of O139 strains (n=2) it was 85-95 per cent and in case of CT-producing non-O1/non-O139 strains it was 90-95 per cent (Fig. 2).

Recent cholera epidemics predominantly caused by the El Tor variant strains in many developing countries is a matter of concern because of the higher pathogenic potential of these variant strains^3^,^34^. One of the reasons could be higher CT production by El Tor variant strains possessing classical *ctx*B gene allele than typical El Tor^35^. The result of our study also showed similar trend, *i.e*., higher CT production among El Tor variant strains^19^. However, our results indicate that the inhibitory impact of capsaicin is not influenced by the amount of CT production by various *V. cholerae* strains. Importantly, capsaicin can effectively inhibit CT production not only in El Tor variants but also in typical El Tor, O139, classical as well as in non-O1/non-O139 strains (Fig. 2)^19^.

## Inhibition of transcription of virulence and regulatory genes of *V. cholerae* by red chili and capsaicin

**Fig. 3:**
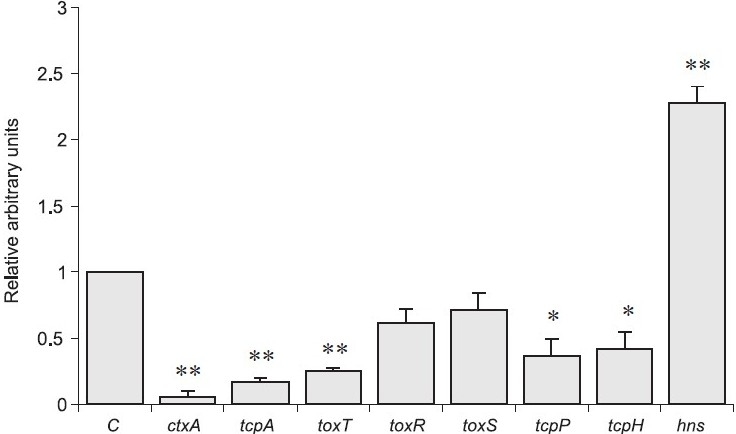
Effect of capsaicin on transcription of various regulatory and virulence genes in *V. cholerae* strain^19^. Transcription of virulence-related genes of *V. cholerae* O1 El Tor variant CRC41 strain was investigated by qRT-PCR assay in the presence of capsaicin (100 μg/ml). The relative transcription level of each gene was compared using *rec*A gene as an internal control. ‘C’ indicates control value of target gene transcription without red chili methanol extract and capsaicin (arbitrarily taken as 1). Statistical significance of the observed differences was calculated using a two-sample *t* test. (*, *P*<0.05; **, *P*<0.01).

Expression of CT and TCP is activated by the expression of ToxT which in turn is regulated by TcpP/TcpH and ToxR/ToxS^36^,^37^. On the contrary, histone-like nucleoid structuring protein (H-NS) encoded by the *hns* gene, a global prokaryotic gene regulator, has been shown to repress the transcription of several virulence genes including *toxT, ctx* and *tcpA*^38^. To analyze the inhibitory mechanisms of red chili or one of its major components capsaicin on CT production in *V. cholerae* strains, quantitative reverse transcription real time PCR (qRT-PCR) analysis was performed using a representative strain belonging to *V. cholerae* O1 El Tor variant. This strain was grown with and without red chili extract or capsaicin. Total RNA was extracted and qRT-PCR was performed following TaqMan probe method. Red chili extract repressed the transcription of *ctxA* gene more than 43-fold (*P*<0.01) whereas capsaicin repressed it about 23-fold (*P*<0.01) (Fig. 3)^19^. The influence of capsaicin (100 μg/ml) was further checked targeting transcription of *tcpA*, *toxT*, *toxR*, *toxS*, *tcpP*, *tcpH* and *hns* genes^19^. Transcription of some of these genes was also repressed by capsaicin, *e.g*., *tcpA* (6.3-fold; *P*<0.01), *toxT* (4.0 fold; *P*<0.01), *tcpP* (2.7 fold; *P*<0.05) and *tcpH* (2.5-fold; *P*<0.05) (Fig. 3). Interestingly, transcription of hns was enhanced more than two times by capsaicin (*P*<0.01)^19^. In the qRT-PCR assay the transcription of *recA*, a housekeeping gene used as control, was not affected in the presence or absence of red chili extract and capsaicin.

**Fig. 4:**
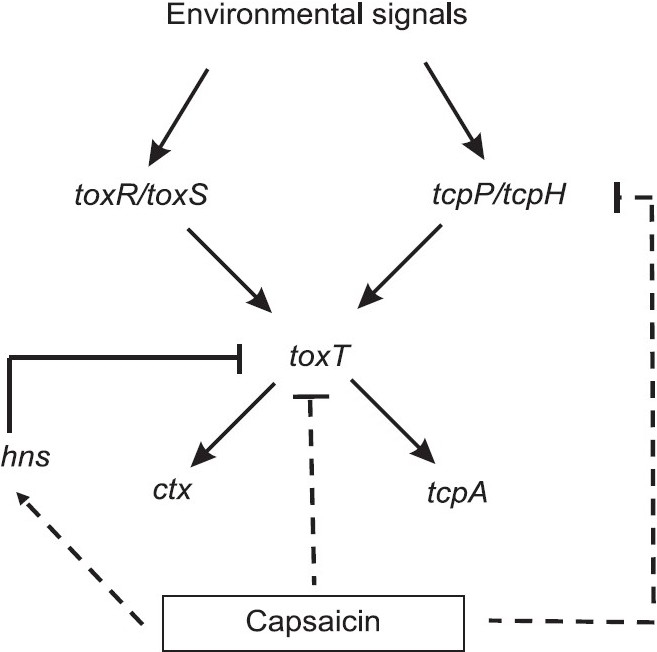
Possible mechanisms of virulence repression of *V. cholerae* by capsaicin^19^. The arrow shows activation of transcription of *toxR/toxS* and *tcpP/tcpH* by environmental signals, which subsequently activates that of *ctx* and *tcpA* through activation of transcriptional activator *toxT*^40^. H-NS is a basal repressor of *toxT, ctx* and *tcpA* under non-permissive condition. Capsaicin directly or indirectly repressed transcription of *toxT, ctx* and *tcpA* but enhanced that of *hns* gene (indicated by dot).

Reduction in the transcription of *ctxA*, *tcpA* and *toxT* genes in the presence of capsaicin may be due to the inhibitory effect of capsaicin on ToxT (Figs. 3 and 4). Previous study has also reported that a synthetic compound virstatin (4-[N-(1,8-naphthalimide)]-n-butyric acid) can inhibit CT production in a ToxT dependent manner^39^. However, it has been shown earlier that H-NS negatively regulates the transcription of *toxT*, *ctx* and *tcpA* genes^38^. Another study has demonstrated that in the presence of bile hns can also repress *ctx* and *tcpA* transcriptions in a ToxT independent manner^30^. As capsacin could enhance hns gene transcription (Fig. 3), it is plausible that hns may play a critical role in the reduction of transcriptions of *ctxA* and *tcpA*^19^. Therefore, capsaicin may directly or indirectly activate the *hns* transcription which in turn downregulates the transcription of *toxT*, *ctxA* and *tcpA* genes (Figs. 3 and 4. Besides, capsaicin may directly repress the transcription of these three genes (Fig. 4). In case of the transcription of *toxR/toxS* regulatory genes capsaicin had no impact but the compound repressed *tcpP/tcpH* transcription (Fig. 3). ToxR is believed to instigate CT production by inducing toxT transcription via synergistic coupling of ToxR/ToxS and TcpP/TcpH^37^. These data suggest that capsaicin could repress transcription of virulence genes via induction of hns in a ToxR-independent manner (Fig. 4). However, red chili extract showed higher inhibitory impact in comparison to capsaicin which indicates the possibility of having other unidentif"ied compound(s) in red chili that can directly inhibit or synergistically act with capsaicin. We have noticed that fatty acid mixture fraction of red chili extract can also inhibit CT production (Fig. 1). Further studies regarding the purification of other active compound(s) present in red chili and other spices extracts as well as *in vivo* study with sub-bacteriocidal concentration of spices extracts are ongoing in our laboratory.

## Conclusion

Various natural compounds can be used to treat cholera in parallel to the conventional therapeutic agents. Particularly, spices like red chili, white pepper and sweet fennel can inhibit CT production in *V. cholerae*. As these spices act against virulence expression rather than the viability of *V. cholerae* there is less chance of developing resistance. One of the active compounds present in red chili is capsaicin, which can inhibit CT production in *V. cholerae* strains regardless of their serogroups and biotypes. The inhibitory mechanism of CT production by capsaicin is probably due to the H-NS mediated inhibition of the transcription of major virulence genes such as *ctx* and *tcpA* genes. Regular intake of commonly available and inexpensive spices (especially, red chili, sweet fennel and white pepper) can be a possible approach to reduce the disease vulnerability from *V. cholerae*.
